# Glutathione S-transferase genotypes modify lung function decline in the general population: SAPALDIA cohort study

**DOI:** 10.1186/1465-9921-8-2

**Published:** 2007-01-11

**Authors:** Medea Imboden, Sara H Downs, Oliver Senn, Gabor Matyas, Otto Brändli, Erich W Russi, Christian Schindler, Ursula Ackermann-Liebrich, Wolfgang Berger, Nicole M Probst-Hensch, the SAPALDIA Team

**Affiliations:** 1 Institutes of Social and Preventive Medicine & Surgical Pathology, Molecular Epidemiology/Cancer Registry, University of Zurich & University Hospital Zurich, Switzerland; 2 Institute of Social and Preventive Medicine, University of Basel, Switzerland; 3 Institute of Medical Genetics, Division of Medical Molecular Genetics and Gene Diagnostics, University of Zurich, Switzerland; 4 Zürcher Höhenklinik, Wald, Switzerland; 5 Department of Pneumology, University Hospital Zurich, Switzerland; 6 Division of Pulmonary Medicine, University Hospitals of Geneva, Switzerland

## Abstract

**Background:**

Understanding the environmental and genetic risk factors of accelerated lung function decline in the general population is a first step in a prevention strategy against the worldwide increasing respiratory pathology of chronic obstructive pulmonary disease (COPD). Deficiency in antioxidative and detoxifying Glutathione S-transferase (GST) gene has been associated with poorer lung function in children, smokers and patients with respiratory diseases. In the present study, we assessed whether low activity variants in GST genes are also associated with accelerated lung function decline in the general adult population.

**Methods:**

We examined with multiple regression analysis the association of polymorphisms in *GSTM1*, *GSTT1 *and *GSTP1 *genes with annual decline in FEV1, FVC, and FEF_25–75 _during 11 years of follow-up in 4686 subjects of the prospective SAPALDIA cohort representative of the Swiss general population. Effect modification by smoking, gender, bronchial hyperresponisveness and age was studied.

**Results:**

The associations of GST genotypes with FEV1, FVC, and FEF_25–75 _were comparable in direction, but most consistent for FEV1. *GSTT1 *homozygous gene deletion alone or in combination with *GSTM1 *homozygous gene deletion was associated with excess decline in FEV1 in men, but not women, irrespective of smoking status. The additional mean annual decline in FEV1 in men with *GSTT1 *and concurrent *GSTM1 *gene deletion was -8.3 ml/yr (95% confidence interval: -12.6 to -3.9) relative to men without these gene deletions. The *GSTT1 *effect on the FEV1 decline comparable to the observed difference in FEV1 decline between never and persistent smoking men. Effect modification by gender was statistically significant.

**Conclusion:**

Our results suggest that genetic *GSTT1 *deficiency is a prevalent and strong determinant of accelerated lung function decline in the male general population.

## Background

According to estimates by the World Health Organization chronic obstructive pulmonary disease (COPD) has become the fourth most common single cause of death and its prevalence is further increasing world wide [1]. COPD is characterized by irreversible and progressive bronchial obstruction and is associated with persistent airway inflammation [2]. Excess age-related lung function decline is considered a subclinical correlate of COPD and is associated with morbidity and premature mortality [3]. The identification of risk factors leading to accelerated lung function decline is thus needed for efficient COPD prevention. Modifiable risk factors for COPD include active cigarette smoking, occupational dust and fume exposure [4] and possibly air pollution [5] and passive smoking [6].

But there is also broad evidence that genetic differences influence the individual's susceptibility to COPD. Rare mutations in the *SERPINA1 *gene [7], leading to severe alpha 1-antitrypsin deficiency, in the *SERPINE2*, protease inhibitor 7 gene, and in the *ELN*, elastin gene, have been identified as genetic predisposing factor in families with early onset COPD [8,9]. To what degree common genetic variants influence susceptibility to COPD in the general population is the focus of intensive research efforts. There is limited evidence from association studies on common genetic polymorphisms in various candidate genes that modify the individual's risk for lung function deficits and COPD [10-13]. But several lines of evidence point to the involvement of the supergene family of glutathione S-transferase (GST) in respiratory disease etiology, including that of COPD. Given their function in the metabolism of environmental toxicants as well as in the inactivation of reactive oxygen species, these genes represent promising candidates for modification of the susceptibility to tobacco-smoke derived and other inhaled irritants [14,15]. Two prevalent homozygous gene deletions of the Mu-1 and Theta-1 GST members (*GSTM1 *and *GSTT1*) have repeatedly been associated with increased susceptibility to respiratory disease and lung function deficits in children, asthmatics, and smokers with respiratory symptoms [16-20]. It is, however, unknown whether these GST polymorphisms also influence lung function in the general population. We therefore investigated the association of the three most studied GST polymorphisms (*GSTM1 *and *GSTT1 *gene deletions and *GSTP1 *Ile105Val single nucleotide polymorphism) with change in lung function over an eleven year follow-up using the population-based SAPALDIA cohort (Swiss Cohort Study on Air Pollutants and Lung and Heart Diseases in Adults) and hypothesized that low-activity variants would also accelerate lung function decline in the general adult population.

## Methods

### Study population

The SAPALDIA cohort study has been described in details elsewhere [21,22]. In brief, participants predominantly of European-Caucasian ethnicity and Swiss nationality, were randomly selected from eight regional population registries [21,22]. Health examinations at baseline (1991) and follow-up (2002) included an interview about respiratory health, occupational and lifestyle exposures as well as spirometry, a methacholine bronchial challenge test and end-expiratory carbon monoxide measurement. Participants gave informed consent at both surveys separately for health examination, interview and blood analysis. The SAPALDIA cohort study complies with the Helsinki Declaration and has received ethical approval by the central ethics committee of the Swiss Academy of Medical Sciences and the Cantonal Ethics Committees for each of the eight examination areas.

Participation rate in SAPALDIA at baseline was 59.3%. Of 9651 participants examined at baseline, 8047 subjects (86%) agreed to participate fully or partially at follow-up. For the present investigation no selection of SAPALDIA participants was made, but we included all subjects with complete information on outcome and covariate data. 5973 subjects (62%) completed the entire follow-up protocol including spirometry and blood sampling. For 275 participants no DNA was available for genetic testing due to refusal or insufficient blood sample volume. Valid spirometry data on FEV1, FVC and FEF_25_-_75 _were not available from both surveys for 215, 310 and 373 participants, respectively. Genotyping for one or more genetic polymorphisms failed in 13 participants. Missing information on one or more covariates included in the regression models further diminished the sample size (n = 784). The final sample size was 4686, 4591 and 4528 subjects for the investigation of annual change in FEV1, FVC and FEF_25_-_75_, respectively. Comparison of the baseline characteristics of SAPALDIA cohort participants included in and excluded from this analysis revealed that excluded SAPALDIA participants were on average older, more likely to have been smokers at baseline examination, and had reported a slightly higher number of pack-years at baseline (Table 1). Accordingly lung function was slightly lower and the proportion of subjects with an FEV1/FVC ratio below 70% was slightly higher in non-participants excluded from this current investigation.

**Table 1:** Baseline characteristics* of the included versus excluded SAPALDIA participants

Variables:	Participants Included n = 4686	Participants Excluded† n = 4965
Women [N/%]	2455/52.4	2453/49.4
Age [years]	40.8 (± 11.5)	41.3 (± 11.8)
BMI at baseline [kg/m2]	23.7 (± 3.6)	24.2 (± 4.1)
		
*Smoking*		
Non-smokers [N/%]	2325/49.6	1904/38.5
Former smokers [N/%]	948/20.2	1227/24.8
Current smokers [N/%]	1413/30.2	1819/36.7
		
Pack-years at baseline ‡	18.4 (± 18.4)	19.6 (± 20.1)
		
*Lung Function*		
FEV1 [L]	3.6 (± 0.8)	3.5 (± 0.9)
FEV1 % pred. §	100.2 (± 13.3)	98.0 (± 14.8)
FEV1/FVC	79.2 (± 7.6)	78.9 (± 8.4)
FEV1/FVC <70%	468/10.1	510/12.0

* expressed as mean (± SD) for quantitative variables and absolute numbers/percentages for categorical variable.

† SAPALDIA participants at the baseline examination who did not participate at follow-up (n = 3678) [21] or who were excluded from the present analysis due to missing information on outcome or covariate data (n = 1287).

‡ mean of pack-years among ever smoking subjects. Participants were defined as ever-smokers if they had smoked at least 20 packs of cigarettes or 360 g of tobacco in their life.

§FEV1 % predicted calculations based on SAPALDIA specific prediction equations [55, 56].

INSERT [Table 1]

### Spirometry and bronchial hyperresponsiveness

The spirometry measurement procedures at both time points have been described elsewhere in detail [21,22]. Briefly, identical spirometer devices (Sensormedics model 2200, Yorba Linda, USA) and protocols were used at baseline and follow-up and their comparability was assessed prior to the follow-up study [23]. Three to maximal eight forced expiratory lung function manoeuvres were performed by each participant and a minimum of two acceptable forced expiratory flows, forced vital capacity (FVC), forced expiratory volume in the first second (FEV_1_) and forced expiratory flows during the middle half of the FVC (FEF_25–75_) complying with American Thoracic Society criteria [24] were obtained. Expiratory flow measures with the highest sum of FVC, FEV_1 _and FEF_25–75 _were taken from the same flow-volume curves.

Bronchial hyperresponsiveness (BHR) to methacholine chloride (Provocholine^®^, Roche, Nutley, New Jersey, USA) was defined as presence of a 20% or greater drop in FEV1 compared to the highest FEV1-value measured during the test. Increasing concentrations of methacholine (0.39, 1.56, 6.25, and 25.0 mg/ml solutions in a phosphate buffer without phenol) were administered through an aerosol dosimeter (Mefar MB3, Bovezzo, Italy) up to a cumulative dose of 2 mg (8.37 ug/mol).

### Genotyping

DNA was extracted from EDTA blood using the PUREGENE™ DNA purification kit (GENTRA Systems, Minneapolis, USA)[21]. In all subjects *GSTM1 *and *GSTT1 *gene deletions and a single nucleotide polymorphism (SNP) in *GSTP1 *leading to the amino acid substitution Ile105Val were genotyped on the ABI Prism 7000 sequence detection system (Applied Biosystems, Rotkreuz, Switzerland) using 5'nuclease real time PCR (TaqMan^®^) assay and fluorescently labeled allele-specific probes. Following primers and probes were used for *GSTM1*: forward 5'-GGACATTTTGGAGAACCAGACC-3' and reverse 5'-CTGGATTGTAGCAGATCATGCC-3' primers and *GSTM1*-specific probe 5'-VIC-TGGACAACCATATGCAG-MGB-3'; for *GSTT1*: forward 5'-GTCATTCTGAAGGCCAAGGACTT-3' and reverse 5'-GGCATCAGCTTCTGCTTTATGGT-3' primers and *GSTT1*-specific probe 5'-FAM-CACCTGCAGACCCC-MGB-3'; for *GSTP1 *Ile105Val: forward 5'-CCTGGTGGACATGGTGAATGAC-3' and reverse 5'-CAGATGCTCACATAGTTGGTGTAGA-3' primers and Ile105 -specific probe 5'-VIC-CTGCAAATACATCTCC-MGB-3'and Val 105 -specific probe 5'-FAM- CTGCAAATACGTCTCC-MGB-3'. *GSTM1*/*GSTT1 *assays were repeated for all DNA samples carrying double homozygous *GSTM1 *and *GSTT1 *deletions using internal positive *GSTP1 *controls. All double homozygous deletion carriers could be confirmed. With this approach, hemizygous *GSTM1 *or *GSTT1 *carriers were not distinguishable from homozygous carriers. In addition a 5% random sample of all DNA samples was regenotyped with highest reproducibility (>99.5%). Hardy-Weinberg equilibrium (HWE) was tested for *GSTP1 *Ile105Val using Arlequin (Version 2.000) software [25].

### Statistical analysis

The dependent variable, annual change in lung function, was calculated by dividing the difference between follow-up and baseline lung function by the number of follow-up years. Multiple linear regression analysis was used to estimate in a fixed effect model of the association of GST genotypes with annual change in lung function. Covariates included in the models were baseline lung function, age, sex, height, weight change during follow-up, study center, level of education, exposure to gas and dust at work at baseline, smoking status at baseline and at follow-up, pack-years smoked at baseline and during follow-up. Cumulative cigarette smoking exposure was summarized in two separate variables: "pack-years smoked up to baseline" and "pack-years smoked during follow-up". The following categories of smoking status were derived for the current study: "Never smokers" reported to be non-smokers at both surveys (n = 2258). "Ever smokers" had to have smoked more than 20 packs of cigarettes or more than 360 g of tobacco (n = 2428) in their lifetime by the end of the follow-up period. Ever smokers were further divided into: "persistent smokers" reported current smoking at both surveys (n = 1026), and "others" were all remaining subjects, comprising participants reporting at both surveys former smoking (n = 944), quitting smoking during follow-up (n = 387), starting smoking during follow-up (n = 38), non-smoking at baseline and former smoking at follow-up (n = 29) and former smoking at baseline and current smoking at follow-up (n = 4). 48 participants provided inconsistent smoking information. Exclusion of these subjects in a sensitivity analysis did not change the strength or the direction of the association observed. Effect modification of genotype/lung function associations by gender, smoking status, and smoking intensity (pack-years up to baseline and during follow-up in ever smokers), as well as BHR and age, was assessed by including according multiplicative interaction terms in the regression models. Trend tests for the combination of *GSTT1 *and *GSTM1 *genotypes were conducted by using a genotype combination variable coded as "presence of zero, one and two gene deletion polymorphisms" as ordinal variable in the model. Two-sided p-values of <0.05 and <0.10 were considered as statistically significant for main effects and interactions [26], respectively. Correction for multiple testing was done using the conservative Bonferroni correction. The associations were corrected for the number of statistical tests performed (main effects and interactions with gender and smoking intensity) (thirty comparisons per lung function parameter investigated, consisting of fifteen tests in men and fifteen tests in women: all; never smokers; persistent smokers). The Bonferroni corrected significance level for the *a priori *hypotheses regarding association between GST genotypes and lung function change in men and women including the *a priori *assessment of interaction with gender and smoking and was P > 0.0017. Sensitivity analyses regarding age and BHR were not corrected for multiple testing. All analyses were conducted using STATA SE version 8.0 (Stata Corporation, TX, USA).

## Results

Characteristics of the study population are summarized in Table 2. The study included more women (52.4%) than men (47.6%). Reflecting recruitment as a random sample of the Swiss general population participants had on average good lung function at baseline and follow-up. FEV1 percent predicted at baseline and follow-up was 100.2% and 97.0% of predicted values, respectively. The mean annual change in FEV1 was -39.6 ml/yr (SD: ± 33.6) in men and -31.8 ml/yr (± 26.2) in women, respectively. Women were more likely to be never smokers. Among smoking subjects, men smoked on average more heavily than women (21.8 pack-years vs. 14.5 pack-years at baseline; 7.1 vs. 5.5 pack-years during follow-up). The observed GST genotype distributions agreed well with previous reports in Caucasians [19,27,28]. *GSTM1 *and *GSTT1 *null genotypes were present in 53% and 18% of all subjects. The homozygous *GSTP1 *Val/Val genotype was present in 9.4% and its allele distribution was in Hardy-Weinberg equilibrium.

INSERT [Table 2]

**Table 2:** Characteristics* of the study population, the SAPALDIA Cohort

	**All**	**Women**	**Men**
N	4686	2455/52.4%	2231/47.6%
Age [years]	40.8 (± 11.5)	41.2 (± 11.4)	40.4 (± 11.6)
BMI at baseline [kg/m2]	23.7 (± 3.6)	22.9 (± 3.8)	24.5 (± 3.2)
BMI at follow-up [kg/m2]	25.8 (± 4.4)	25.2 (± 4.8)	26.5 (± 3.8)
Weight change			
during follow-up [kg]	5.6 (± 6.2)	5.5 (± 6.1)	5.7 (± 6.3)
			
*Smoking †*			
Never smokers	2258/48.2	1354/55.2	904/40.5
Ever smokers	2428/51.8	1101/44.8	1327/59.5
Persistent smokers	1026/21.9	487/19.8	539/24.2
Others	1402/29.9	614/25.0	788/35.3
Pack-years up to baseline ‡	18.4 (± 18.4)	14.5 (± 14.5)	21.8 (± 20.6)
Pack-years during follow-up ‡	6.4 (± 8.5)	5.5 (± 6.5)	7.1 (± 9.8)
			
*Lung Function*			
FEV1 at baseline [L]	3.6 (± 0.8)	3.1 (± 0.6)	4.1 (± 0.7)
FVC at baseline [L]	4.5 (± 1.1)	3.8 (± 0.6)	5.3 (± 0.8)
FEF25–75 at baseline [L]	3.4 (± 1.2)	3.1 (± 1.0)	3.8 (± 1.3)
Annual change FEV1 [ml/yr]	-35.5 (± 30.2)	-31.8 (± 26.2)	-39.6 (± 33.6)
Annual change FVC [ml/yr]	-24.2 (± 41.0)	-20.6 (± 34.9)	-28.3 (± 46.5)
Annual change FEF25–75 [ml/yr]	-71.3 (± 65.4)	-68.6 (± 59.4)	-74.1 (± 71.2)
FEV1 % pred. at baseline §	100.2 (± 13.3)	100.8 (± 13.4)	99.4 (± 13.2)
FEV1 % pred. at follow-up §	97.0 (± 14.4)	98.6 (± 14.1)	95.4 (± 14.6)
FEV1/FVC at baseline [%]	79.2 (± 7.6)	80.4(± 7.4)	78.0 (± 7.7)
FEV1/FVC at follow-up [%]	74.8 (± 7.5)	75.5 (± 7.1)	74.0 (± 7.8)
FEV1/FVC at follow-up <70%	1030/22.0	473/19.3	557/25.0
Positive BHR at baseline [%]	612/16.1	394/20.3	218/11.7
			
*Genotypes*			
*GSTM1 *deletion	2477/52.9	1306/53.2	11171/52.5
*GSTT1 *deletion	844/18.0	466/19.0	378/17.0
*GSTP1 *Ile105Val			
Ile/Ile	2219/47.4	1162/47.3	1057/47.4
Ile/Val	2025/43.2	1068/43.5	957/42.9
Val/Val	442/9.4	225/9.2	217/9.7

* expressed as mean (± SD) for quantitative variables and absolute numbers/percentages for categorical variable.

† "Never" smokers reported non-smoking at both surveys. "Persistent" smokers reported current smoking at both surveys. "Others" comprised participants reporting at both surveys former smoking (n = 944), quitting smoking during follow-up (n = 387), starting smoking during follow-up (n = 38), non-smoking at baseline and former smoking at follow-up (n = 29) and former smoking at baseline and current smoking at follow-up (n = 4).

‡ mean of pack-years among ever smoking subjects. Ever-smokers encompass both, persistent smokers and others.

§FEV1 % predicted calculations based on predicted values from SAPALDIA specific prediction equations [55, 56].

### Association of GST genotypes with lung function decline

No independent association of *GSTM1 *or *GSTP1 *genotype with any of the lung function parameters was observed, irrespective of gender. *GSTT1 *gene deletion alone or in combination with *GSTM1 *deletion was associated with accelerated lung function decline in men, but not women. Men homozygous for the *GSTT1 *gene deletion exhibited an excess annual change in FEV1 of -5.3 ml/yr (P = 0.001). The *GSTT1 *effects on FVC and FEF_25–75 _were comparable in size and direction, but did not reach statistical significance. Men carrying the double homozygous gene deletions of *GSTT1 *and *GSTM1 *had on average a -8.3 ml/yr greater annual decline in FEV1 than men with at least one copy of both, the *GSTT1 *and the *GSTM1 *gene (P for trend <0.001); the according excess change was -6.5 ml/yr (P = 0.045) for FVC and -7.8 ml/yr (P = 0.094) for FEF_25–75_. The interactions between gender and *GSTT1 *deletion alone or in combination with *GSTM1 *deletion were statistically significant for FEV1, FEF_25–75 _and FVC (for *GSTT1*/*GSTM1 *combination only).

INSERT [Table 3]

**Table 3:** Adjusted* associations of GST genotypes† with excess annual decline in FEV1, FVC and FEF_25–75 _stratified by sex, the SAPALDIA Cohort.

	**Difference in mean annual change in lung function (ml/yr) **‡											
	**FEV1 (ml/yr)**				**FVC (ml/yr)**				**FEF**_25–75 _**(ml/yr)**			
	**n**	**Coeff.**	**95%CI**	**p-value **§	**n**	**Coeff.**	**95%CI**	**p-value **§	**n**	**Coeff.**	**95%CI**	**p-value **§
**MEN**												
GSTT1 non-null	1853	---		---	1806	---		---	1787	---		---
GSTT1 null	378	-5.3	-8.4, -2.1	0.001¶**	371	-5.2	-7.4, -1.3	0.17	366	-5.0	-11.7, 1.8	0.15
												
GSTM1 non-null	1060	---		---	1040	---		---	1030	---		---
GSTM1 null	1171	-2.1	-4.5, 0.3	0.081	1137	-0.8	-4.0, 2.5	0.65	1123	-4.1	-9.2, 0.9	0.11
												
GSTP1 105 Ile/Ile	1057	---		---	1037	---		---	1025	---		---
GSTP1 105 Ile/Val	957	0.09	-2.4, 2.6	0.94	929	-0.1	-3.5, 3.3	0.95	919	-2.7	-8.0, 2.6	0.32
GSTP1 105 Val/Val	217	-2.5	-6.6, 1.7	0.25	211	-3.2	-9.0, 2.5	0.27	209	-7.3	-16.1, 1.6	0.11
												
GSTM1T1 both non-null	885	---		---	866	---		---	860	---		---
GSTM1T1 either null	1143	-1.8	-4.3, 0.7	---	1114	0.9	-2.5, 4.4	---	1097	-5.1	-10.5, 0.2	---
GSTM1T1 both null	203	-8.3	-12.6, -3.9	<0.001¶**	197	-6.5	-12.5, -0.5	0.045¶	196	-7.8	-17.0, 1.5	0.094
GSTT1*GSTM1Interaction				0.30				0.042¶				0.55
												
**WOMEN**												
GSTT1 non-null	1989	---		---	1953	---		---	1920	---		---
GSTT1 null	466	-0.3	-2.5, 1.8	0.76	462	-1.6	-4.5, 1.3	0.28	455	3.4	-1.5, 8.3	0.18
												
GSTM1 non-null	1149	---		---	1131	---		---	1115	---		---
GSTM1 null	1306	0.5	-1.2, 2.2	0.54	1284	1.1	-1.2, 3.4	0.36	1260	-0.8	-4.7, 3.1	0.69
												
GSTP1 105 Ile/Ile	1162	---		---	1142	---		---	1126	---		---
GSTP1 105 Ile/Val	1068	-0.6	-2.4, 1.2	0.51	1050	0.6	-1.8, 3.0	0.64	1030	-3.0	-7.0, 1.1	0.15
GSTP1 105 Val/Val	225	-0.2	-3.3, 2.9	0.90	223	1.5	-2.6, 5.6	0.48	219	0.5	-6.5, 7.5	0.88
												
GSTM1T1 both non-null	931	---		---	915	---		---	902	---		---
GSTM1T1 either null	1276	-0.9	-2.7, 1.0	---	1254	-1.1	-3.5, 1.4	---	1231	-0.6	-4.8, 3.5	---
GSTM1T1 both null	248	1.6	-1.5, 4.6	0.24	246	1.3	-2.7, 5.4	0.42	242	3.2	-3.6, 10.0	0.53
GSTT1*GSTM1Interaction				0.026¶				0.025¶				0.66
Sex* Genotype Interaction††												
sex*GSTT1				0.010¶				0.56				0.043¶
sex*GSTM1				0.028¶				0.23				0.19
sex*GSTP1				0.50				0.39				0.40
sex*GSTT1M1				<0.001¶				0.015¶				0.08¶

* The effects of GST genotypes are adjusted for the respective baseline lung function parameter, smoking status at baseline and follow-up, pack-years smoked at baseline and during follow-up, height, weight change between surveys, study area, gas and dust exposure at baseline and education level.

† *GSTM1 *and *GSTT1 *genotypes were dichotomized into absence vs. presence of homozygous gene deletions (non-null vs. null). The effect of the *GSTP1 *genotype on lung function change was investigated in a co-dominant genetic model with the Ile/Ile genotype as the reference group. The combined *GSTM1 */*GSTT1 *genotype (GSTM1T1) was coded as "presence of zero, one and two homozygous gene deletion polymorphisms" and included as ordinal variable in the linear regression model.

‡ Change in lung function parameter represented the difference between lung function parameter measured at follow-up [ml] and the one measured at baseline [ml] divided by the duration of follow-up period [yr]. Coefficient values below zero correspond to an excess decline in lung function [ml/yr] compared to the decline in the reference group and coefficient values above zero correspond to a less steep decline in lung function compared of the reference group.

§Uncorrected P-values for differences between categories. Bonferroni corrected significance level for multiple comparisons: P < 0.0017.

¶Statisically significant (uncorrected P-value > 0.05). ** Statistically significant after Bonferoni-correction (P-value > 0.0017)

†† Interaction between genotype and gender was assessed by including interaction terms in the model.

The majority of the reported association results did not withhold the conservative Bonferroni correction; however the *GSTT1 *genotype alone or in combination with *GSTM1 *genotype showed a significant association with annual change in FEV1 even after Bonferroni correction. The effect of double homozygous *GSTT1 *and *GSTM1 *deletion on lung function decline is graphically presented as predicted mean annual FEV1 decline in different genotype/gender strata.

INSERT [Figure 1]

**Figure 1:**
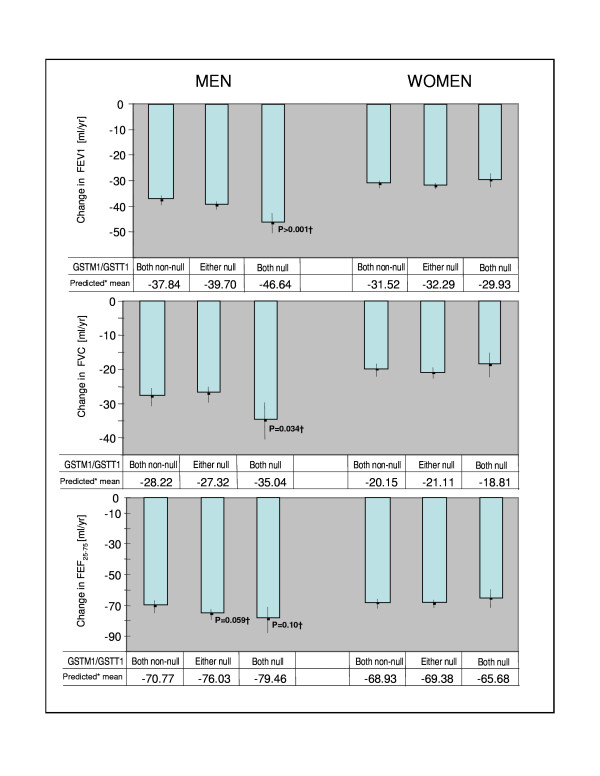
Predicted* mean annual change in lung function parameter by the combined *GSTT1 *and *GSTM1 *gene deletion genotype and sex, the SAPALDIA cohort. * adjusted for baseline FEV1, FVC and FEF_25–75_, respectively, as well as for smoking status at baseline and follow-up, pack-years smoked at baseline and during follow-up, height, weight change between surveys, study area, gas and dust exposure at baseline and education level. † P-values for difference with reference group GSTM1T1 both non-null.

### Effect modification by smoking

An important determinant of premature lung function decline is active smoking. In our study population, persistent male smokers exhibited on average an -6.6 ml/yr greater annual FEV1 change than never smoking men; the average change in FEV1 was -42.8 ml/yr (± 35.6) in male persistent smokers and -36.2 ml/yr (± 33.2) in male never smokers. In persistent smokers each pack-year smoked during follow-up was associated with an excess average annual FEV1 change of -0.8 ml/yr. We assessed the impact of genetic GST deficiency on lung function decline separately for never smokers and persistent smokers; associations observed in ever-smokers were similar to those reported here for persistent smokers (data not shown). Irrespective of gender or smoking status no independent effects of *GSTM1 *or *GSTP1 *Ile105Val genotype on accelerated decline of FEV1, FVC or FEF_25–75 _were observed. Male persistent smokers with *GSTT1 *null genotype exhibited on average an excess annual decline in FEV1 of -8.0 ml/yr (P = 0.013) when compared to persistent smokers with *GSTT1 *non-null genotype. The according *GSTT1 *effect in male never smokers was -5.6 ml/yr (P = 0.025). The difference in *GSTT1 *effect between persistent and never smokers was not statistically significant (P for interaction>0.10). The *GSTT1 *effect in persistent smokers was modified by packyears smoked to baseline (P for interaction<0.001) and during follow-up (P for interaction = 0.029). Similar trends for the GSTT1 effect on FEV1 decline in smoking strata, though lacking statistical significance, were observed for FVC and less clearly for FEF_25–75_. The *GSTT1 *genotype alone or in combination with *GSTM1 *genotype was not associated with excess lung function change in women, irrespective of smoking status. There was a suggestion that heterozygosity for the *GSTP1 *Ile105Val SNP was associated with slower decline in FVC in persistent smokers (P = 0.030), but no according heterozygous effects on FEV1 or FEF_25–75 _were observed.

INSERT [Table 4]

**Table 4:** Adjusted* associations of GST genotypes† with excess annual decline in FEV1, FVC and FEF_25–75 _stratified by smoking status and sex, the SAPALDIA Cohort.

**MEN**	**Difference in mean annual change in lung function (ml/yr) **‡											
	**FEV1 (ml/yr)**				**FVC (ml/yr)**				**FEF**_25–75 _**(ml/yr)**			
**Male Never Smokers**	**n**	**Coeff.**	**95%CI**	**p-value **§	**n**	**Coeff.**	**95%CI**	**p-value **§	**n**	**Coeff.**	**95%CI**	**p-value **§
GSTT1 non-null	750	---		---	731	---		---	723	---		---
GSTT1 null	154	-5.6	-10.6, -0.7	0.025¶	150	-1.0	-7.8, 5.7	0.76	148	-8.5	-19.0, 1.9	0.11
												
GSTM1 non-null	432	---		---	424	---		---	420	---		---
GSTM1 null	472	-2.4	-6.1, 1.4	0.22	457	-1.8	-6.8, 3.3	0.50	451	-4.4	-12.3, 3.5	0.27
												
GSTP1 105 Ile/Ile	433	---		---	426	---		---	420	---		---
GSTP1 105 Ile/Val	389	-0.2	-4.2, 3.7	0.90	377	-0.1	-5.5, 5.2	0.96	375	-4.3	-12.5, 4.0	0.31
GSTP1 105 Val/Val	82	-2.0	-8.8, 4.7	0.56	78	-5.9	-15.1, 3.3	0.21	76	-4.5	-18.9, 9.9	0.54
												
GSTM1T1 both non-null	351	---		---	344	---		---	342	---		---
GSTM1T1 either null	480	-2.2	-6.2, 1.7	---	467	0.9	-4.4, 6.3	---	459	-4.8	-13.1, 3.5	---
GSTM1T1 both null	73	-9.7	-16.9, -2.5	0.029¶	70	-7.1	-16.9, 2.7	0.26	70	-14.4	-29.6, 0.78	0.15
												
	**FEV1 (ml/yr)**				**FVC (ml/yr)**				**FEF25–75 (ml/yr)**			
**Male Persistent Smokers**	**n**	**Coeff.**	**95%CI**	**p-value **§	**n**	**Coeff.**	**95%CI**	**p-value **§	**n**	**Coeff.**	**95%CI**	**p-value **§

GSTT1 non-null	428	---		---	417	---		---	410	---		---
GSTT1 null	111	-8.0	-14.2, -1.7	0.013¶	109	-9.3	-17.6, -1.1	0.027¶	106	-3.7	-16.9, 9.5	0.58
												
GSTM1 non-null	256	---		---	249	---		---	244	---		---
GSTM1 null	283	-4.2	-9.3, 1.0	0.11	277	-4.1	-10.8, 2.7	0.24	272	-6.1	-16.9, 4.7	0.27
												
GSTP1 105 Ile/Ile	258	---		---	252	---		---	246	---		---
GSTP1 105 Ile/Val	228	0.2	-5.3, 5.6	0.95	222	-4.6	-11.7, 2.5	0.20	218	4.7	-6.6, 16.0	0.41
GSTP1 105 Val/Val	53	-1.0	-10.0, 7.9	0.82	52	-1.0	-12.7, 10.7	0.87	52	-4.7	-23.1, 13.7	0.62
												
GSTM1T1 both non-null	207	---		---	200	---		---	197	---		---
GSTM1T1 either null	270	-4.9	-10.4, 0.5	---	266	-6.6	-13.8, 0.6	---	260	-5.8	-17.3, 5.7	---
GSTM1T1 both null	62	-11.5	-20.1, -2.9	0.022¶	60	-11.5	-22.8, -0.2	0.072	59	-9.3	-27.3, 8.8	0.48
Genotype*Packyears Interaction ††												
GSTT1*during follow-up				0.029¶				0.67				0.89
GSTT1*up to baseline				0.001¶				0.02¶				0.41
												
GSTM1*during follow-up				0.50				0.30				0.33
GSTM1*up to baseline				0.39				0.70				0.83
												
GSTP1*during follow-up				0.96				0.37				0.65
GSTP1*up to baseline				0.31				0.84				0.32
												
GSTM1T1*during follow-up				0.75				0.11				0.40
GSTM1T1*up to baseline				0.31				0.34				0.88

**WOMEN**	**Difference in mean annual change in lung function (ml/yr) **‡											
	**FEV1 (ml/yr)**				**FVC (ml/yr)**				**FEF25–75 (ml/yr)**			

**Female Never Smokers**	**n**	**Coeff.**	**95%CI**	**p-value **§	**n**	**Coeff.**	**95%CI**	**p-value **§	**n**	**Coeff.**	**95%CI**	**p-value **§
GSTT1 non-null	1091	---		---	1072	---		---	1053	---		---
GSTT1 null	263	-0.7	-3.6, 2.3	0.66	260	-1.7	-5.7, 2.3	0.40	256	0.5	-6.2, 7.2	0.88
												
GSTM1 non-null	629	---		---	617	---		---	607	---		---
GSTM1 null	725	0.5	-1.8, 2.8	0.67	715	0.9	-2.2, 4.1	0.56	702	-0.7	-6.0, 4.6	0.79
												
GSTP1105Ile/Ile	644	---		---	631	---		---	621	---		---
GSTP1105Ile/Val	582	-1.2	-3.6, 1.2	0.32	575	-1.4	-4.7, 1.9	0.41	564	-2.5	-8.0, 3.1	0.38
GSTP1105Val/Val	128	-0.8	-4.9, 3.3	0.69	126	0.3	-5.3, 5.9	0.91	124	1.0	-8.5, 10.4	0.84
												
GSTM1T1 both non-null	506	---		---	496	---		---	488	---		---
GSTM1T1 either null	708	-1.5	-3.9, 1.0	---	697	-0.6	-4.0, 2.7	---	684	-3.8	-9.5, 1.9	---
GSTM1T1 both null	140	1.8	-2.3, 5.9	0.20	139	0.44	-5.1, 6.0	0.89	137	3.3	-5.9, 12.6	0.19
												
	**FEV1 (ml/yr)**				**FVC (ml/yr)**				**FEF25–75 (ml/yr)**			
**Female Persistent Smokers**	**n**	**Coeff.**	**95%CI**	**p-value **§	**n**	**Coeff.**	**95%CI**	**p-value **§	**n**	**Coeff.**	**95%CI**	**p-value **§

GSTT1 non-null	428	---		---	385	---		---	376	---		---
GSTT1 null	111	4.8	-0.3, 9.8	0.065	92	3.5	-3.1, 10.2	0.30	92	12.7	1.7, 23.6	0.029¶
												
GSTM1 non-null	256	---		---	230	---		---	227	---		---
GSTM1 null	283	1.2	-2.9, 5.2	0.57	247	1.8	-3.6, 7.1	0.51	241	-0.5	-9.4, 8.3	0.91
												
GSTP1 105 Ile/Ile	258	---		---	227	---		---	223	---		---
GSTP1 105 Ile/Val	228	1.9	-2.3, 6.0	0.38	214	6.0	0.6, 11.5	0.03¶	210	-2.1	-11.1, 7.0	0.66
GSTP1 105 Val/Val	53	4.3	-3.4, 12.1	0.27	36	3.0	-7.2, 13.3	0.56	35	10.5	-6.5, 27.5	0.23
												
GSTM1T1 both non-null	207	---		---	194	---		---	191	---		---
GSTM1T1 either null	270	3.2	-1.1, 7.4	---	227	1.5	-4.1, 7.2	---	221	5.8	-3.6, 15.1	---
GSTM1T1 both null	62	4.0	-2.7, 10.7	0.27	56	5.3	-3.5, 14.1	0.50	56	7.4	-7.1, 21.8	0.40
												
Genotype*Packyears Interaction ††				**Persistent**				**Persistent**				**Persistent**
GSTT1*during follow-up				0.23				0.75				0.09¶
GSTT1*up to baseline				0.20				0.52				0.19
												
GSTM1*during follow-up				0.79				0.58				0.44
GSTM1*up to baseline				0.69				0.72				0.58
												
GSTP1*during follow-up				0.33				0.065				0.50
GSTP1*up to baseline				0.80				0.41				0.44
												
GSTM1T1*during follow-up				0.67				0.82				0.81
GSTM1T1*up to baseline				0.68				0.88				0.82

* The effects of GST genotypes are adjusted for the respective baseline lung function parameter, smoking status at baseline and follow-up, pack-years smoked at baseline and during follow-up, height, weight change between surveys, study area, gas and dust exposure at baseline and education level.

† *GSTM1 *and *GSTT1 *genotypes were dichotomized into absence vs. presence of homozygous gene deletions (non-null vs. null). The effect of the *GSTP1 *genotype on lung function change was investigated in a co-dominant genetic model with the Ile/Ile genotype as the reference group. The combined *GSTM1 */*GSTT1 *genotype (GSTM1T1) was coded as "presence of zero, one and two homozygous gene deletion polymorphisms" and included as ordinal variable in the linear regression model.

‡ Change in lung function parameter represented the difference between lung function parameter measured at follow-up [ml] and the one measured at baseline [ml] divided by the duration of follow-up period [yr]. Coefficient values below zero correspond to an excess decline in lung function [ml/yr] compared to the decline in the reference group and coefficient values above zero correspond to a less steep decline in lung function compared of the reference group.

§Uncorrected P-values for differences between categories. Bonferroni corrected significance level for multiple comparisons: P < 0.0017.

ll Interaction between pack-years and genotype in smokers was assessed by including interaction terms in the models. Cumulative cigarette smoking exposure was summarized in two separate variables: "pack-years smoked up to baseline" and "pack-years smoked during follow-up".

¶Statisically significant (uncorrected P-value > 0.05).

### Sensitivity analysis: modification of the GST effects by BHR

The GST genotypes have previously been associated with asthma and BHR [28]. Restriction of the analysis to subjects without a report of asthma (data not shown) and without the presence of BHR at either baseline or follow-up (Table 5) revealed comparable associations in size between GST genotypes and lung function change as reported for the whole study population (Table 3), irrespective of gender and lung function parameter. Thus the observed GST/lung function decline associations are not merely due to an effect of GST on asthma or BHR.

**Table 5:** Adjusted* associations of GST genotypes† with excess annual decline in FEV1, FVC and FEF_25–75 _in men and women, stratified by absence or presence of BHR at baseline or follow-up, the SAPALDIA Cohort.

**BHR**	**Difference in mean annual change in lung function (ml/yr) **‡											
**negative**	**FEV1 (ml/yr)**				**FVC (ml/yr)**				**FEF25–75 (ml/yr)**			
	**n**	**Coeff.**	**95%CI**	**p-value **§	**n**	**Coeff.**	**95%CI**	**p-value **§	**n**	**Coeff.**	**95%CI**	**p-value **§
**MEN**												
GSTT1 non-null	965	---		---	948	---		---	941	---		---
GSTT1 null	209	-4.4	-8.3, -0.5	0.027¶	208	-3.8	-9.0, 1.3	0.14	207	-4.2	-13.2, 4.8	0.36
												
GSTM1 non-null	560	---		---	550	---		---	546	---		---
GSTM1 null	614	-0.8	-3.8, 2.2	0.58	606	0.2	-3.7, 4.2	0.91	602	-3.4	-10.4, 3.5	0.33
												
GSTP1 105 Ile/Ile	557	---		---	547	---		---	542	---		---
GSTP1 105 Ile/Val	511	0.9	-4.1, 2.2	0.56	505	-0.7	-4.9, 3.4	0.95	503	-4.3	-11.6, 2.9	0.24
GSTP1 105 Val/Val	106	-0.6	-6.0, 4.9	0.84	104	-0.7	-7.9, 6.6	0.86	103	-3.7	-16.3, 9.0	0.57
												
GSTM1T1 both non-null	460	---		---	450	---		---	447	---		---
GSTM1T1 either null	605	-0.5	-3.7, 2.7	---	598	0.7	-3.5, 4.9	---	593	-3.2	-10.5, 4.1	---
GSTM1T1 both null	109	-6.4	-11.8, -1.0	0.062	108	-5.1	-12.3, 2.1	0.27	108	-8.0	-20.5, 4.5	0.41
												
	**FEV1 (ml/yr)**				**FVC (ml/yr)**				**FEF25–75 (ml/yr)**			
	**n**	**Coeff.**	**95%CI**	**p-value **§	**n**	**Coeff.**	**95%CI**	**p-value **§	**n**	**Coeff.**	**95%CI**	**p-value **§
**WOMEN**												
GSTT1 non-null	812	---		---	808	---		---	803	---		---
GSTT1 null	181	1.2	-1.8, 4.1	0.43	181	0.3	-3.9, 4.5	0.88	180	5.4	-2.0, 12.9	0.15
												
GSTM1 non-null	459	---		---	458	---		---	456	---		---
GSTM1 null	534	0.7	-1.6, 3.0	0.55	531	0.2	-3.0, 3.5	0.88	527	2.1	-3.7, 7.8	0.48
												
GSTP1 105 Ile/Ile	472	---		---	470	---		---	469	---		---
GSTP1 105 Ile/Val	430	-0.6	-2.4, 1.2	0.51	428	0.5	-2.9, 3.9	0.76	425	-5.6	-11.6, 0.5	0.072
GSTP1 105 Val/Val	91	-0.2	-3.3, 2.9	0.90	91	4.3	-1.6, 10.2	0.15	89	-3.6	-14.1, 6.9	0.50
												
GSTM1T1 both non-null	373	---		---	372	---		---	370	---		---
GSTM1T1 either null	525	-1.5	-3.9, 0.9	---	522	-1.1	-4.6, 2.3	---	519	4.4	-1.7, 10.6	---
GSTM1T1 both null	95	0.1	-4.1, 4.3	0.24	95	2.3	-3.6, 8.1	0.46	94	5.5	-5.0, 15.9	0.31

**BHR**	**Difference in mean annual change in lung function (ml/yr) **‡											
**positive**	**FEV1 (ml/yr)**				**FVC (ml/yr)**				**FEF25–75 (ml/yr)**			
	**n**	**Coeff.**	**95%CI**	**p-value **§	**n**	**Coeff.**	**95%CI**	**p-value **§	**n**	**Coeff.**	**95%CI**	**p-value **§

**MEN**												
GSTT1 non-null	276	---		---	267	---		---	265	---		---
GSTT1 null	51	-5.5	-14.7, 3.8	0.24	50	-5.5	-17.3, 6.3	0.36	50	-7.0	-24.2, 10.2	0.42
												
GSTM1 non-null	165	---		---	161	---		---	161	---		---
GSTM1 null	162	-8.2	-14.9, -1.5	0.017¶	156	-2.8	-11.4, 5.8	0.53	154	-12.4	-24.9, 0.08	0.051
												
GSTP1 105 Ile/Ile	142	---		---	139	---		---	137	---		---
GSTP1 105 Ile/Val	146	-0.7	-8.0, 6.6	0.84	139	-6.0	-15.3, 3.3	0.20	139	7.8	-5.8, 21.4	0.26
GSTP1 105 Val/Val	39	0.07	-10.8, 10.9	0.99	39	-2.8	-16.4, 10.9	0.69	39	-2.9	-22.8, 17.0	0.77
												
GSTM1T1 both non-null	139	---		---	136	---		---	136	---		---
GSTM1T1 either null	163	-6.1	-13.2, 1.0	---	156	-0.2	-9.3, 8.8	---	154	-11.6	-24.8, 1.6	---
GSTM1T1 both null	25	-17.3	-30.6, .4,1	0.024¶	25	-13.4	-30.2, 3.4	0.27	25	-20.2	-44.6, 4.3	0.12
												
Among men: interaction Genotype* BHR ††												
GSTT1*BHR				0.71				0.89				0.65
												
GSTM1*BHR				0.33				0.94				0.66
												
GSTP1*BHR				0.44				0.84				0.24
												
GSTM1T1*BHR				0.57				0.99				0.81
												
	**FEV1 (ml/yr)**				**FVC (ml/yr)**				**FEF25–75 (ml/yr)**			
	**n**	**Coeff.**	**95%CI**	**p-value **§	**n**	**Coeff.**	**95%CI**	**p-value **§	**n**	**Coeff.**	**95%CI**	**p-value **§

**WOMEN**												
GSTT1 non-null	437	---		---	428	---		---	423	---		---
GSTT1 null	101	-0.8	-5.8, 4.3	0.77	100	-1.1	-7.6, 5.4	0.74	99	0.8	-9.2, 10.9	0.87
												
GSTM1 non-null	251	---		---	246	---		---	244	---		---
GSTM1 null	287	0.6	-3.4, 4.6	0.76	282	1.0	-4.2, 6.1	0.72	278	0.9	-7.1, 8.8	0.83
												
GSTP1 105 Ile/Ile	250	---		---	246	---		---	243	---		---
GSTP1 105 Ile/Val	250	-2.4	-6.6, 1.7	0.24	244	-1.8	-7.1, 3.4	0.50	242	-5.5	-13.7, 2.7	0.19
GSTP1 105 Val/Val	38	2.2	-5.8, 10.2	0.59	38	5.3	-4.9, 15.6	0.31	37	-6.3	-22.2, 9.6	0.43
												
GSTM1T1 both non-null	206	---		---	202	---		---	200	---		---
GSTM1T1 either null	276	-1.5	-7.8, 2.8	---	270	-1.5	-7.0, 3.9	---	267	-2.8	-11.3, 5.7	---
GSTM1T1 both null	56	1.9	-5.0, 8.8	0.56	56	2	-6.8, 10.8	0.68	55	5.4	-8.4, 19.1	0.46
												
Among women: interaction Genotype* BHR ††												
GSTT1*BHR				0.25				0.23				0.52
												
GSTM1*BHR				0.85				0.67				0.47
												
GSTP1*BHR				0.77				0.87				0.35
												
GSTM1T1*BHR				0.41				0.85				0.24

* The effects of GST genotypes are adjusted for the respective baseline lung function parameter, smoking status at baseline and follow-up, pack-years smoked at baseline and during follow-up, height, weight change between surveys, study area, gas and dust exposure at baseline and education level.

† *GSTM1 *and *GSTT1 *genotypes were dichotomized into absence vs. presence of homozygous gene deletions (non-null vs. null). The effect of the *GSTP1 *genotype on lung function change was investigated in a co-dominant genetic model with the Ile/Ile genotype as the reference group. The combined *GSTM1 */*GSTT1 *genotype (GSTM1T1) was coded as "presence of zero, one and two homozygous gene deletion polymorphisms" and included as ordinal variable in the linear regression model.

‡ Change in lung function parameter represented the difference between lung function parameter measured at follow-up [ml] and the one measured at baseline [ml] divided by the duration of follow-up period [yr]. Coefficient values below zero correspond to an excess decline in lung function [ml/yr] compared to the decline in the reference group and coefficient values above zero correspond to a less steep decline in lung function compared of the reference group.

§Uncorrected P-values for differences between categories.

¶Statistically significant (uncorrected P-value > 0.05).

†† Interaction between genotype and BHR was assessed by including interaction terms in the model.

BHR was previously shown to be predictive of COPD [29]. Results of the investigation of the GST effects on decline in lung function among BHR positive subjects (Table 5) suggested that the respective impact of *GSTT1 *and *GSTM1 *gene deletion might be modified by BHR. The interaction between GST genotypes and BHR did not reach statistical significance, though. In male BHR positive subjects, *GSTM1 *rather then *GSTT1 *deficiency was associated with accelerated decline in FEV1 (-8.2 ml/yr, P = 0.017) and FEF_25–75 _(-12.4 ml/yr, P = 0.051). Again, the lung function decline was strongest for the combined *GSTM1/GSTT1 *genotypes, consistent with a gene dose-response. For both, FEV1 and FEF_25–75 _effect estimates for GSTM1T1 both null were stronger than those observed among male BHR negative subjects. No association of GST genotype with FVC was observed in male BHR positive subjects. In BHR positive women again no statistically significant GST genotype/lung function associations were observed.

INSERT [Table 5]

### Sensitivity analysis: GST effect in age restricted subpopulation

Both, lung function growth and decline are age-dependent processes. The SAPALDIA cohort also includes young adults (age at baseline 18 to 60 years). To confirm that the observed associations between GST genotype and lung function change are due to an impact of these genotypes on age-related decline, we restricted analysis to subjects older than 30 years, an age at which lung growth has ceased and age-related lung function decline started [30] (data not presented). In men we observed associations of *GSTT1 *alone or in combination with *GSTM1 *with change in FEV1 and FVC that were similar in trend to the ones observed in the entire study sample (for *GSTT1 *and FEV1: -5.8 ml/yr (P = 0.001); for *GSTM1 *and *GSTT1 *both null and FEV1: -7.4 ml/yr (P = 0.009)). The association with change in FVC was more pronounced (for *GSTT1*: -4.4 ml/yr (P = 0.08); for *GSTM1 *and *GSTT1 *both null: -9.5 ml/yr (P = 0.005)). In contrast, the non-significant association observed for *GSTT1 *genotype and change in FEF_25–75 _was no longer present in men aged 30 years or older (for *GSTT1*: -0.4 ml/yr (P = 0.89); for *GSTM1 *and *GSTT1 *both null: -1.9 ml/yr (P = 0.72)). Instead, the association between *GSTM1 *null genotype and excess annual change in FEF_25–75 _became statistically significant (for *GSTM1*: -7.6 ml/yr (P = 0.024)). In women over age 30 at baseline, we did not observe any GST genotype/lung function decline association.

## Discussion

Our results suggest that genetic *GSTT1 *deficiency alone or in combination with *GSTM1 *deficiency is independently associated with an accelerated age-related decline of lung function in men, but not women, irrespective of smoking status. The impact size of the *GSTT1 *genotype was comparable to the difference in FEV1 decline that we observed between male persistent smokers and never smokers.

This is the first study reporting an association between GST genotypes and lung function in the general adult population. Genetically determined GST deficiency has previously been associated with deficits in lung function growth and respiratory symptoms in healthy and asthmatic children exposed to oxidative inhalants such as high ambient ozone concentrations and passive smoke, respectively [20,31]. While GSTs are well known for their role in the metabolism of exogenous toxic substrates including tobacco derived substances, they also exhibit peroxidase activity and thus might play an important role in oxidative stress defense [15]. The fundamental relevance of the oxidative stress pathway to respiratory health and disease is evidenced by the fact that dietary and circulating antioxidants have been suggested by a number of epidemiological studies to protect the lung from accelerated pulmonary function decline and other respiratory diseases [32-34]. The current observation that the GST genotype effects were even present in never smokers living in study areas with moderate concentrations of ambient ozone and other air pollutants is in line with this notion and with experimental data suggesting that various air pollutants as well as chronic inflammatory processes can cause oxidative damage to the lung tissue at low levels [35].

The *GSTT1 *effect was most consistent for FEV1 and overall comparable in direction for FVC and FEF_25–75 _in our study. Yet our results suggest that the respective impacts of GST genotypes on the different lung function parameters may depend on the smoking status, BHR and age. The strength of the *GSTT1 *association with FVC and FEF_25–75 _differed between never smokers and persistent smokers. The effect on change in FEF_25–75 _was not observed in persistent smokers as also reflected by the lack of interaction of smoking history (pack-years) with *GSTT1 *genotype, suggesting that the *GSTT1 *affects predominately large airway caliber in smokers. In addition no statistically significant *GSTT1 *effect on any lung function parameter was observed in subjects with bronchial hyperresponsiveness. Accelerated decline in FEV1 has been well characterized to correlate with development of COPD [36], but measures of small airways function such as changes in FEF_25–75 _have also been reported to be of relevance in COPD [37]. Air pollutant exposure to ozone [38] and NO2 [39] has often been associated with a greater decline in FEF_25–75 _than in FEV1. Differences in age, as well as differences in genotypes investigated between our participants and previous study populations may explain in part the inconsistency in the GST effects on various lung function parameters. Alterations in FEF_25–75 _and FEF_25–75_/FVC have been interpreted as indicators of airway-parenchymal dysanaptic lung growth. These parameters may be of greater relevance as an outcome for GST and oxidant effects during childhood. Ongoing research into genetic determinants of various lung function parameters may provide further insight into the biology of different lung function parameters [40].

A novel finding of our population-based study is the pronounced gender difference in the association between GST genotypes and age-related change in lung function. Though the biological basis of these observed gender differences is unknown, gender differences in lung function and respiratory diseases have consistently been observed throughout life. They can be attributed in part to sex-specific immunological and hormonal patterns associated with lung function [41]. In addition, men and women seem to differ in susceptibility to exogenous exposures. On one hand comparative studies suggest that women recover better than men from the adverse effects of tobacco smoke [42-44]. On the other hand there is evidence that women had greater respiratory deficits per pack-year smoked [45] as well as higher DNA adducts levels when adjusted for cigarette dose [46]. Several other lines of evidence indicate in contrast that women might be more resistant to oxidative stress than men. In smokers, air flow obstruction was reported to be more strongly associated with the presence of high-grade preinvasive epithelial lesion in men than in women [47]. Women compared to men were found to exhibit increased systemic antioxidant capacities such as higher glutathione blood levels [48], increased glutathione peroxidase activity [49] and less oxidant-damaged DNA at advanced age [50,51]. In addition, women often report increased antioxidant intake when compared to men, which may make them less receptive for an effect of low penetrance gene variants [34]. Finally, recently reported evidence suggests that the estrogen receptor is involved in the up-regulation of oxidative stress defensive genes including *GSTP1 *[52] supporting the notion that sex-specific mechanisms in the defense response to oxidative stress exist. If confirmed by additional studies, our results which point to a sex-specific GST impact on lung function might be indicative of a broad biological basis for gender difference in susceptibility to airborne toxicants.

We observed in the male SAPALDIA population differences in the relative impact of specific GST polymorphisms on lung function. In fact, results from previous studies suggest that the respective relevance of GST genes on respiratory health may depend on age, health and exposure status of the study population [16,18,19,53,54]. The *GSTT1 *gene deletion polymorphism has previously been identified as an important determinant of age-related lung function change [19,53]. In the Lung Health Study (LHS) the rate of lung function decline was accelerated in *GSTT1 *deficient smokers with mild COPD [19,53]. No gender difference was observed, but women were underrepresented in the LHS. The *GSTT1 *effect was stronger in mild as opposed to heavy smokers, whereas in our study the *GSTT1 *effect was more pronounced in smokers and with increasing pack-years. This discrepancy to the LHS results might be attributable to the comparatively better respiratory health state of the SAPALADIA participants (FEV1 % pred. [55,56] at follow-up: 97%) and to their more moderate smoking habits. The relative impact of specific GST genotypes on lung function in children may be different from that in adults. Gilliland et al. [20] reported lower lung function growth in children with *GSTP1 *105Val and *GSTM1 *null genotypes, but not with *GSTT1 *null genotype. In agreement with our results, no independent *GSTM1 *effect on pulmonary function was observed in healthy, non-smoking freshmen students, irrespective of their antioxidant intake [34]. Exclusion of younger age groups in our study population did not modify the reported associations, demonstrating that the findings on association between GST genotypes and lung function change were mostly due to the genotype impact on age related lung function decline rather than on lung function growth. As suggested by our sensitivity analysis the relative impact of *GSTT1 *or *GSTM1 *gene deletion may also depend on the presence or absence of BHR. There is evidence that BHR responsive airways are more vulnerable to oxidative particles and to airway inflammation in general [57]. This altered physiology of the BHR positive lung tissue might provide a pathophysiologic basis for the respective impact of *GSTM1 *deficiency reported here.

Results are most inconsistent with regard to the *GSTP1 *Ile105Val genotype. The *GSTP1 *105Val allele was found to be protective against asthma and BHR [54]. In the LHS study *GSTP1 *105Val/Val genotype was associated with lower lung function at baseline as well as with more rapid lung function decline in smokers with high baseline lung function values [19]. Yet the combination of *GSTM1 *and *GSTT1 *gene deletion and *GSTP1 *105Ile/Ile was defined as risk genotype in the follow-up study of the LHS [53]. In other studies, the *GSTP1 *105Ile/Ile genotype was inconsistently associated with COPD [13,54,58]. In vitro assays further underline the complexity of the functional impact of the *GSTP1 *Ile105Val polymorphism, since the relative activity of the variants is substrate dependent [59]. *GSTP1*, the most abundantly expressed member of the GST gene family in the lung [60], may have a complex impact on respiratory disease. *GSTP1 *appears to act not only as a detoxifying and antioxidative enzyme, but also as direct inhibitor of the C-Jun N terminal kinase [61]. Accordingly, low *GSTP1 *expression or activity has been reported to promote apoptosis in lung epithelium [54,62]. Future studies investigating more comprehensively genetic variation and haplotypes of the *GSTP1 *gene should improve our understanding of the role of *GSTP1 *at various developmental and phenotypic levels of respiratory health.

The strength of the SAPALDIA cohort is its prospective design, its large sample size as well as the detailed characterization of study participants. However, several limitations of the study deserve to be discussed. First, the study cohort was evaluated at two single time points eleven years apart and the range of factors influencing lung function decline were assessed through personal interviews depending on reporting/recalling bias of the study participants. Also there is the concern for selection bias for participation at follow-up. Comparison of baseline characteristics of SAPALDIA participants included in this investigation with SAPALDIA cohort participants not included in this analysis due to missing covariate information suggested that the population sample investigated here represents a younger, and less actively smoking and healthier sample. GST genotypes were not associated with age or smoking behavior among subjects included in this study. Since it is not likely that GST genotypes influenced study participation, non-participation at baseline, loss to follow-up and exclusion of participants due to lacking covariate information is unlikely to invalidate the results presented. Second, a hypothetical limitation of our association study may be potential population stratification since the Swiss population consists of multiple language groups. Deviation of HWE of the *GSTP1 *Ile105Val genotype was not observed within the three language groups presented in our study (French, German and Italian); the genotype distribution was comparable in the three language groups. The prevalence of gene deletion genotypes of *GSTM1 *and *GSTT1 *and were not statistically significantly different by language region or nationality. Neither language group nor Swiss nationality did modify the observed associations between GST deficient genotypes and lung function decline. Given the low power of HWE [63], genotype data from additional unlinked genetic markers should ideally be used for testing population admixture [64]. However limited funding prohibited this control of population stratification in our study. Nevertheless we do not expect population stratification in this Swiss cohort to invalidate the observed associations since genetic homogeneity of Caucasian Western-Central European populations [65] has been repeatedly described. A further limitation of the study is the fact that the genetic analysis chosen does not permit to disentangle heterozygotes from homozygotes wild type *GSTT1 *or *GSTM1 *genotypes. It is conceivable that even stronger associations with lung function could have been observed in a contrast of subjects without any deletion allele versus no *GSTT1 *and *GSTM1 *gene. Finally sample size was limited for the assessment of GST genotype effects among BHR positive subjects.

In conclusion our results suggest that common genetic polymorphisms can influence the rate of lung function decline in the general population. A large proportion of the Caucasian population carry one or both GST gene deletions (~20% of *GSTT1 *gene deletion, ~50% the GSTM1 gene deletion and ~10% of *GSTT1/GSTM1 *gene deletion carriers). The high prevalence and the strong effect size, which is comparable to the effect of smoking, underscore the public health relevance of our results. Additional studies need to confirm and identify the biological mechanisms underlying the newly observed gender difference in GST genotype effects on age-related lung function decline.

## Abbreviations

BHR – Bronchial hyperresponsiveness

BMI – Body Mass Index

COPD – chronic obstructive pulmonary disease

DNA – desoxyribonucleic acid

EDTA – ethylenediaminetetraacetic acid

FEV1 – forced expiratory volume in one second

FVC – forced vital capacity

GST – Glutathione S-transferase

HWE – Hardy-Weinberg equilibrium

Ile – isoleucine

LHS – Lung Health Study

PCR – polymerase chain reaction

SAPALDIA – Study on Air Pollution And Lung Disease In Adults

Val – valine

## Competing interests

The author(s) declare that they have no competing interests.

## Authors' contributions

NMPH conceived the specific research question and designed the SAPALDIA biological sample collection. MI and NMPH established the SAPALDIA DNA bank. OS, MI performed genotype analysis. GM, WB gave major infrastructure support and technical advice for DNA bank establishment and large scale genotyping. NMPH, OB, EWR obtained funding. NMPH, CS, OB, EWR are involved in SAPALDIA Cohort Study and UAL was the co-principal investigator of the SAPALDIA Cohort Study. SHD, CS, MI, OS did the health data management. MI, NMPH, SHD performed data analysis. SHD, CS gave statistical support. All authors contributed to the interpretation of the data and gave critical review during manuscript process. MI and NP drafted the manuscript and all authors read and approved the final manuscript.
